# Neutrophil to Lymphocyte Ratio as a Predictor of Poor Prognosis in Metastatic Pancreatic Cancer Patients Treated with Nab-Paclitaxel plus Gemcitabine: A Propensity Score Analysis

**DOI:** 10.1155/2018/2373868

**Published:** 2018-06-10

**Authors:** J. Ventriglia, A. Petrillo, M. Huerta Alváro, M. M. Laterza, B. Savastano, V. Gambardella, G. Tirino, L. Pompella, A. Diana, F. Iovino, T. Troiani, E. Martinelli, F. Morgillo, M. Orditura, A. Cervantes, F. Ciardiello, F. De Vita

**Affiliations:** ^1^Uro-Gynaecological Department, INT G. Pascale, Via Mariano Semmola, 80131 Naples, Italy; ^2^U.O.C. Medical Oncology, School of Medicine, Università della Campania L. Vanvitelli, c/o II Policlinico, Via Pansini 5, 80131 Naples, Italy; ^3^Department Hematology and Medical Oncology, INCLIVA Biomedical Research Institute, University of Valencia, Blasco Ibáñez 17, 46010 Valencia, Spain; ^4^Breast Department, INT G. Pascale, Via Mariano Semmola, 80131 Naples, Italy; ^5^Department of Cardiothoracic and Respiratory Sciences, School of Medicine, Università della Campania L. Vanvitelli, c/o II Policlinico, Via Pansini 5, 80131 Naples, Italy

## Abstract

**Background:**

High neutrophil to lymphocyte ratio (NLR) has shown to be a predictor of poor outcomes in various malignancies, including pancreatic cancer.

**Methods:**

We assessed 70 consecutive pts with histologically confirmed mPC who received chemotherapy with nab-paclitaxel/gemcitabine at two different European oncologic centers between January 2012 and November 2015. Variables assessed for prognostic correlations included age ≥ 66, sex, Karnofsky PS score, primary tumor site, baseline CA19.9 level ≥ 59xULN, 12-week decrease of the CA19.9 level ≥ 50% from baseline, basal bilirubin level, baseline NLR, biliary stent implantation, and liver metastasis. Survival analyses were generated according to the Kaplan-Meier method. Univariate and multivariate analyses were performed by a Cox proportional hazard model.

**Results:**

According to NLR values, the patients were divided into two groups: high and low. Low group patients showed a better median PFS (7 months versus 5 months) and median OS (13 months versus 7 months) in respect to high group patients. At multivariate analysis, Karnofsky PS < 80% (HR = 0.4; CI 0.2–1.2), liver metastases (HR = 0.4; CI 0.18–0.82), and NLR ≥ 5 (HR = 2.7; 95% CI 1.4–5.2) were predictors of poorer OS. Based on the presence of one or more independent prognostic factors, three risk categories were identified: good-risk, intermediate-risk and poor-risk. The median OS was 22, 10, and 7 months, respectively.

**Conclusions:**

Baseline NLR is an independent predictor of survival of patients with mPC receiving palliative chemotherapy and could be useful to develop a simple clinical score to identify a subgroup of patients with a low chance to benefit from chemotherapy.

## 1. Background

Pancreatic cancer is the ninth most common cancer and the forth cause of cancer-related mortality worldwide. Five-year overall survival (OS) does not exceed 5% due to the fact that more than 85% of patients are diagnosed with incurable locally advanced or metastatic disease [1, 2]. FOLFIRINOX (oxaliplatin, irinotecan, fluorouracil, and leucovorin) or gemcitabine plus albumin-bound paclitaxel is the current standard of care in the first-line setting for patients with metastatic disease [3, 4]. Despite these newer regimens have increased survival, it remains extremely poor still today. Therefore, it is important to clarify the biological mechanisms that contribute to tumor progression as well as to identify prognostic factors for stratify individual risk. During last years, tumor size, histologic grade, vascular invasion, perineural invasion, lymph node metastases, and distant metastases have been recognized as prognostic factors [5–7]. Recently, growing interest in the role of inflammatory response has emerged. Tumor microenvironment is known to have an important role in cancer development and progression and may be associated with systemic inflammation that could be a significant predictor of survival. Hypoalbuminemia, elevated C-reactive protein, increased levels of cytokines, and high leukocyte count and their subtypes are measurable parameters in blood that reflect the systemic inflammatory response [8–13]. Actually, several evidences suggesting that an elevated peripheral blood neutrophil to lymphocyte ratio (NLR) is related to a worse outcome in various types of cancer, including renal cell carcinoma, soft tissue sarcoma, nonsmall cell lung cancer (NSCLC), breast cancer, and colorectal cancer (CRC) [14–22]. We analyzed retrospectively the prognostic independent role of pretreatment NLR in a cohort of 70 metastatic pancreatic patients treated with gemcitabine plus nab-paclitaxel as first-line chemotherapy enrolled in two different European oncologic centers. In this study, we showed that NLR is an independent predictor of the prognosis for metastatic pancreatic cancer patients and that high NLR levels are associated with a short life expectancy.

## 2. Patients and Methods

70 patients were diagnosed with metastatic pancreatic cancer in the Department of Oncology at the Second University of Naples and in the Department of Oncology at the University Hospital in Valencia between January 2012 and December 2015. Written informed consent was obtained from each patient involved in the study, and the research was approved by the Ethic Committee of Second University of Naples. Patients with active infections, hematological disorders or malignancies, or autoimmune disorders, or treated with steroids were excluded from our analysis. Patients with prior adjuvant gemcitabine treatment were included in our analysis only if the treatment was completed at least 6 months before. Data were censored on December 2015. The characteristics of the series are summarized in Table 1. Neutrophil and lymphocyte counts were obtained in peripheral blood before starting chemotherapy and were calculated by dividing the absolute neutrophil count by the absolute lymphocyte count. The cut-off for the NLR was 5 on the basis literature results (NLR < 5, NLR ≥ 5) [23–25]. All patients received first-line chemotherapy with nab-paclitaxel, 125 mg/m^2^, followed by gemcitabine 1000 mg/m^2^ administered intravenously (IV) on days 1, 8, and 15 every 4 weeks until disease progression or evidence of unacceptable toxicity. Recombinant human granulocyte colony-stimulating factor and erythropoietin were administered as needed. Dose reductions were applied in cases of grade III/IV toxicities.

Tumor assessment was performed every 12 weeks according to our clinical practice [26]. OS was defined as the interval between the start of nab-paclitaxel and gemcitabine first-line therapy to death or last follow-up visit. The progression-free survival (PFS) was defined as the interval between the start of nab-paclitaxel and gemcitabine therapy to clinical progression or death or last follow-up visit if not progressed. Variables assessed for prognostic correlations included age ≥ 66, sex, Karnofsky performance status (PS) score, primary tumor site, baseline CA19.9 level ≥ 59xULN, 12-week decrease of the CA19.9 level ≥ 50% from baseline, basal bilirubin level, baseline neutrophil to lymphocyte ratio (NLR), biliary stent implantation, and the presence of liver metastasis. Finally, a prognostic scoring index was planned using the independent prognostic factors identified at multivariate analysis.

## 3. Statistical Analysis

The primary end-point of this analysis was to evaluate the role of prognostic factors on the median OS. Statistical analysis was performed using SPSS 21.0 statistical software. Associations between clinical and histopathological parameters with OS and PFS were investigated using Kaplan-Meier curves and compared by the log-rank test. The chi-square (*χ*^2^) test was used to analyze the relationship between NLR and clinicopathological parameters. Cox regression was applied to multivariate survival analysis to determine effects of probable prognostic factors on PFS and OS. Survival distribution was estimated by the Kaplan-Meier method with 95% confidence interval (CI), and a significant difference was considered when *p* < 0.05 [27]. To adjust for selection bias, all available variables (age ≥ 66, sex, Karnofsky PS score, primary tumor site, baseline CA19.9 level ≥ 59xULN, 12-week decrease of the CA19.9 level ≥ 50% from baseline, basal bilirubin level, biliary stent implantation, and liver metastasis) were introduced in a multivariate logistic regression to calculate a propensity score for each patient with statistical analysis system software (STATA 11). The matching methods used in propensity score analysis are 1 : 1 matching.

## 4. Results

**Figure 1:**
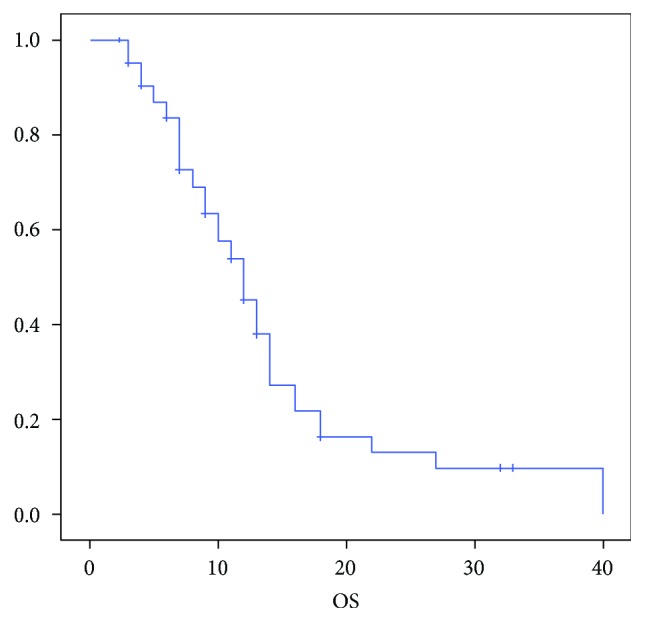
Median OS.

**Figure 2:**
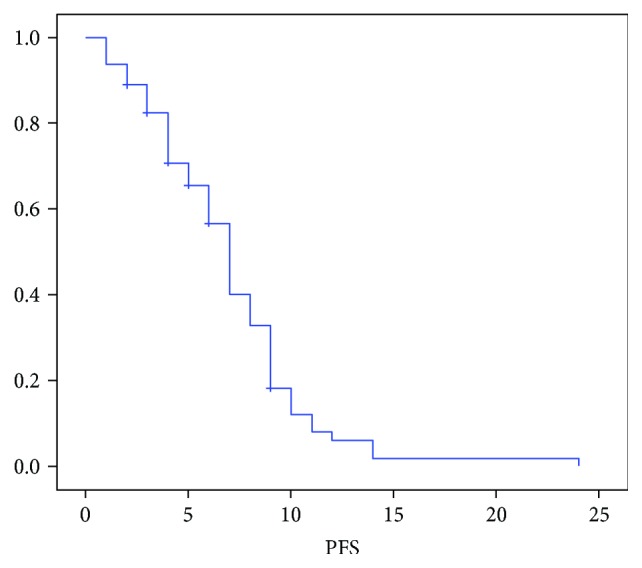
Median PFS.

At the time of data censoring, 56 pts had progression of disease or died; median PFS was 7 months (95% CI 6.221–7.779), and median OS was 12 months (95% CI 9.926–14.074) with a 12-month OS rate of 34.3% (Figures 1 and 2). Three pts (4.3%) were still alive at 24 months after starting chemotherapy.

**Figure 3:**
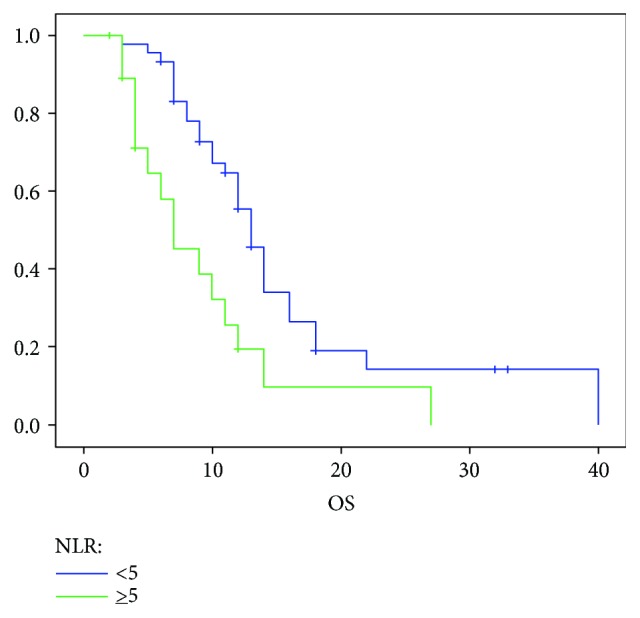
Median OS according to the NLR.

The patients were divided into two groups according to the cut-off of 5: high (*n* = 49) and low NLR (*n* = 21). With a median follow-up of 32 months, median OS of the high NLR groups and low NLR groups was 7 months and 13 months, respectively (*p* = 0.003) (Figure 3). Univariate analysis identified Karnofsky PS (*p* = 0.005), liver metastasis (*p* = 0.01), and NLR ≥ 5 (*p* = 0.005) as significant prognostic factors for poor OS. The Cox multivariate model showed NLR ≥ 5 as an independent prognostic factor (hazard ratio (HR) = 2.7; 95% CI 1.4–5.2; *p* = 0.003) together with Karnofsky PS (HR = 0.4; CI 0.2–1.2; *p* = 0.04) and liver metastasis (HR = 0.4; CI 0.18–0.82; *p* = 0.01) (Table 2).

To further confirm the results observed, a propensity score-matched analysis was performed. Twenty-eight couples were matched and were observed that the NLR ≥ 5 group showed an increase death risk of 19% in respect to the NLR < 5 group (*p* = 0.02).

**Figure 4:**
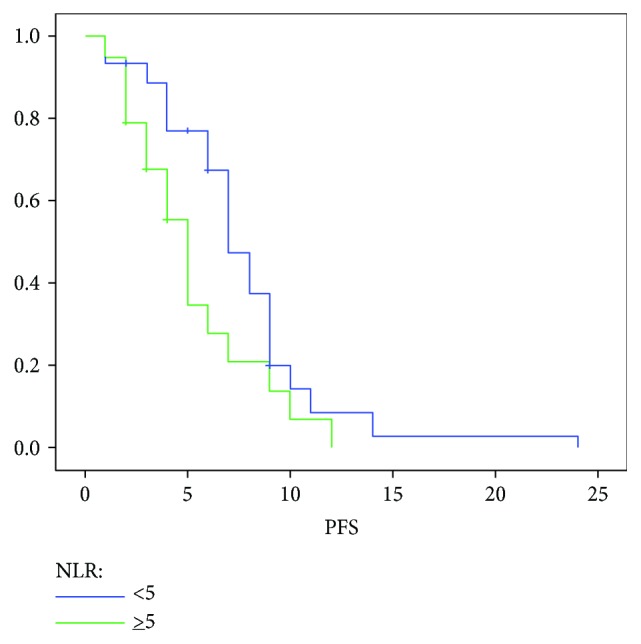
Median PFS according to the NLR.

The PFS in the high NLR group was significantly shorter in respect to the low NLR group (5 versus 7 months; *p* = 0.02; Figure 4). Univariate analysis identified CA19.9 response, the presence of liver metastasis, Karnofsky performance status score, and NLR as significant prognostic factors for high PFS. In the multivariate regression analysis, CA19.9 response and NLR confirmed as independent prognostic factors for high PFS (Table 3).

**Figure 5:**
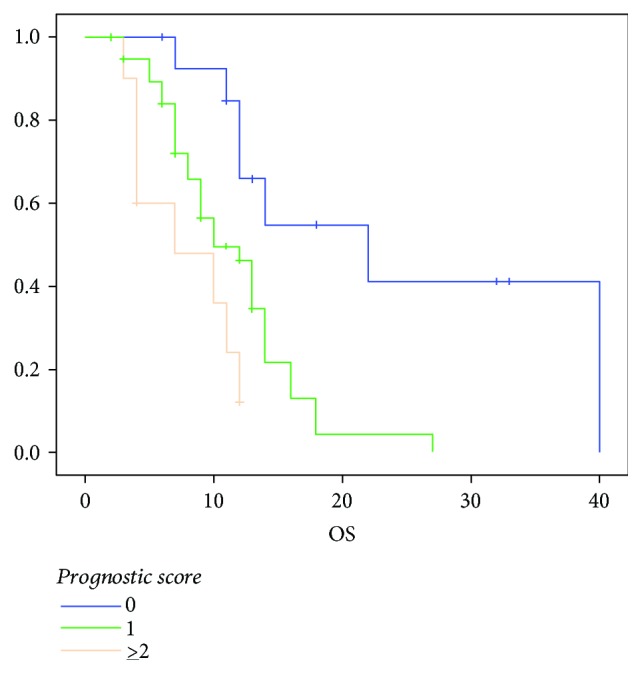
Median OS according to the prognostic score.

Subsequently, we designed a prognostic model based on the independent prognostic factors related to OS: performance status, NLR, and liver metastasis. Based on these parameters, the study population was divided into three risk groups according to the number of independent prognostic factors involved: 0 factor → good prognosis; 1 factor → intermediate prognosis; and 2 factors → poor prognosis. The survival differed notably according to the risk stratification. Patients (*n* = 22) without risk factors (good prognosis) had a median OS of 22 months (95% CI: 8.8–35); patients (*n* = 39) with the presence of one factor (intermediate prognosis) had a median OS of 10 months (95% CI: 6.8–13), and patients (*n* = 9) with the presence of two or more factors (poor prognosis) had a median OS of 7 months (95% CI: 1–15) (Figure 5).

60%, 35.8%, and 22.2% of patients with good, intermediate, and poor prognosis were alive at 12 months, respectively. Furthermore, 20% of patients with good prognosis was alive at 24 months.

## 5. Discussion

Several studies have shown that elevated NLR is associated with worse prognosis in patients with different solid tumors. A recent meta-analysis showed that elevated NLR predicted a poor prognosis in patients with pancreatic cancer (28). These findings are consistent with our results. Using a NLR cut-off of 5, we found that elevated NLR was associated with decreased OS; indeed, patients with NLR ≥ 5 showed a median OS of 7 months compared to 12 months of patients with NLR < 5. Therefore, our results show that NLR ≥ 5 predicts a shorter OS, suggesting that elevated NLR could be a potential biomarker to identify patients with a poor outcome. The relation between NLR and worse prognosis is still unclear and under investigation. It is well known that inflammation contributes to cancer development and progression and that high neutrophil count is a hallmark of systemic inflammation. In particular, neutrophils secreting inflammatory cytokines such as interleukin-2 (IL-2), interleukin-6 (IL-6), interleukin-10 (IL-10), tumor necrosis factor *α* (TNF-*α*), and proangiogenic factors including vascular endothelial growth factor (VEGF) provide a favorable tumor microenvironment for cancer progression. Furthermore, the increased IL-10 and TNF-*α* levels lead to a decrease in the lymphocyte count, a crucial component of innate immunity and the adaptive immune response, with a relevant role in the immune surveillance process towards cancer cells. Therefore, a high NLR could indicate an increased neutrophil-dependent inflammatory response and a reduced lymphocyte-mediated antitumor immune response, in turn able to promote tumor invasiveness, thus resulting in tumor progression and poor outcome [9–11]. Furthermore, we carried out a propensity score matching to adjust for differences in baseline data; this analysis confirmed our results, thus excluding possible selection bias.

According to the results of the IMPACT study and the subsequent analyses [28], we confirm that in a population of metastatic pancreatic cancer patients treated with gemcitabine and nab-paclitaxel, Karnofsky performance status score, the presence of liver metastases, and baseline NLR were independent predictors of survival associated with an increased risk of death. Recently, Goldstein et al. showed that in advanced pancreatic cancer patients treated with FOLFOXIRI, ECOG PS, liver metastases, and NLR were the most important predictors of survival [29]. Furthermore, they categorized this series as good-risk (0 factors), intermediate-risk (1 factor), and poor-risk (≥2 factors), observing significant differences in terms of OS for these 3 groups. Using this prognostic score in a distinct population treated with a different first-line regimen, we have confirmed its usefulness allowing the identification of a subgroup of patients with particularly poor outcome that does not seem to benefit from chemotherapy. Indeed, in our experience the poor-risk group exhibited a median OS of 7 months.

This study has several strengths: all patients presented metastatic disease and received the same chemotherapy regimen as first-line therapy; furthermore, the prognostic meaning of NLR was validated by a propensity score analysis to exclude potential selection bias. On the contrary, the main limitation was represented by the retrospective nature of the study with a small number of patients.

## 6. Conclusion

Our data confirm that elevated baseline NLR is associated with a poor prognosis in patients with metastatic pancreatic cancer treated with gemcitabine and nab-paclitaxel; in addition, the adopted prognostic scoring system might be useful to identify a subgroup of patients with poor prognosis who do not benefit from chemotherapy.

## Figures and Tables

**Table 1:** 

Characteristic	Nab-paclitaxel plus gemcitabine (range)
Age (range)	66 (41–77)
≥70	17 (24%)
Sex	
M	31 (44%)
F	39 (56%)
PS (Karnofsky)	
100%	24 (34%)
80–90%	37 (53%)
60–70%	9 (13%)
Pancreatic primary location	
Head	43 (61%)
Body/tail	27 (39%)
Site of metastasis	
Lung	14 (20%)
Node	7 (10%)
Liver	47 (67%)
Peritoneum	15 (21%)
Bone	3 (4%)
Brain	1 (1%)
Number of metastatic sites	
1	52 (74%)
≥2	17 (26%)
Biliary stent	16 (23%)
Median CA19.9	500 U/I (<1–61,564)
Previous surgery	16 (23%)
Previous adjuvant gemcitabine	10 (14%)

**Table 2:** Univariate and multivariate analysis OS.

Variable	Univariate			Multivariate		
	HR	95% CI	*p* value	HR	95% CI	*p* value
Age (≥66 years versus <66 years)	0.7	0.4–1.3	0.27			
Gender (female versus male)	1.3	0.71–2.41	0.38			
Tumor location (nonhead versus head)	1.3	0.7–2.4	0.4			
Karnofsky performance status score (100–80% versus 70–60%)	0.3	0.12–0.69	**0.005**	0.4	0.2–1.2	0.04
CA19.9 basal (≥59 ULN versus <59 ULN)	1	0.5–2.2	0.8			
CA19.9 reduction (<50% versus ≥50%)	1.8	0.89–3.62	0.098			
Stent (yes versus no)	0.58	0.29–1.16	0.12			
N/L ratio (≥5 versus <5)	2.47	1.3–4.7	**0.005**	2.70	1.4–5.2	**0.003**
Liver metastasis	0.4	0.2–0.8	**0.01**	0.4	0.18–0.82	**0.01**

**Table 3:** Univariate and multivariate analysis PFS.

Variable	Univariate			Multivariate		
	HR	95% CI	*p* value	HR	95% CI	*p* value
Age (≥66 years versus <66 years)	0.86	0.5–1.5	0.58			
Gender (female versus male)	1.1	0.6–1.9	0.71			
Tumor location (nonhead versus head)	1.3	0.7–2.2	0.37			
Karnofsky performance status score (100–80% versus 70–60%)	0.5	0.2–1	0.08			
CA19.9 basal (≥59 ULN versus <59 ULN)	0.8	0.4–1.5	0.5			
CA19.9 reduction (<50% versus ≥50%)	2.44	1.3–4.6	**0.006**	3.22	1.6–6.4	**0.001**
Stent (yes versus no)	0.69	0.37–1.29	0.24			
N/L ratio (≥5 versus <5)	1.9	1.0–3.4	**0.036**	2.77	1.3–5.7	**0.006**
Liver metastasis	0.5	0.3–0.9	**0.038**	0.5	0.2–1.0	0.05

## Data Availability

An anonymized clinical dataset containing all variables used for all analyses of this retrospective study is available from the corresponding author on reasonable request.

## Abbreviations

mPC: Metastatic pancreatic cancer
NLR: Neutrophil to lymphocyte ratio
OS: Overall survival
NSCLC: Nonsmall cell lung cancer
CRC: Colorectal cancer
PFS: Progression-free survival
PS: Performance status
CI: Confidence interval
HR: Hazard ratio
IL-2: Interleukin-2
IL-6: Interleukin-6
IL-10: Interleukin-10
TNF-*α*: Tumor necrosis factor *α*
VEGF: Vascular endothelial growth factor.

## References

[1] Siegel R. L., Miller K. D., Jemal A. (2016). Cancer statistics, 2016. *CA: A Cancer Journal for Clinicians*.

[2] Sant M., Allemani C., Santaquilani M. (2009). EUROCARE-4. Survival of cancer patients diagnosed in 1995–1999. Results and commentary. *European Journal of Cancer*.

[3] Conroy T., Desseigne F., Ychou M. (2011). FOLFIRINOX versus gemcitabine for metastatic pancreatic cancer. *The New England Journal of Medicine*.

[4] Von Hoff D. D., Ervin T., Arena F. P. (2013). Increased survival in pancreatic cancer with nab-paclitaxel plus gemcitabine. *New England Journal of Medicine*.

[5] Vincent A., Herman J., Schulick R., Hruban R. H., Goggins M. (2011). Pancreatic cancer. *Lancet*.

[6] Bilici A. (2014). Prognostic factors related with survival in patients with pancreatic adenocarcinoma. *World Journal of Gastroenterology*.

[7] Tabernero J., Chiorean E. G., Infante J. R. (2015). Prognostic factors of survival in a randomized phase III trial (MPACT) of weekly nab-paclitaxel plus gemcitabine versus gemcitabine alone in patients with metastatic pancreatic cancer. *The Oncologist*.

[8] Mantovani A., Allavena P., Sica A., Balkwill F. (2008). Cancer-related inflammation. *Nature*.

[9] Elinav E., Nowarski R., Thaiss C. A., Hu B., Jin C., Flavell R. A. (2013). Inflammation-induced cancer: crosstalk between tumours, immune cells and microorganisms. *Nature Reviews Cancer*.

[10] Grivennikov S. I., Greten F. R., Karin M. (2010). Immunity, inflammation, and cancer. *Cell*.

[11] O'Callaghan D. S., O'Donnell D., O'Connell F., O'Byrne K. J. (2010). The role of inflammation in the pathogenesis of non-small cell lung cancer. *Journal of Thoracic Oncology*.

[12] Mei Z., Liu Y., Liu C. (2014). Tumour-infiltrating inflammation and prognosis in colorectal cancer: systematic review and meta-analysis. *British Journal of Cancer*.

[13] Lin G., Liu Y., Li S. (2016). Elevated neutrophil-to-lymphocyte ratio is an independent poor prognostic factor in patients with intrahepatic cholangiocarcinoma. *Oncotarget*.

[14] Sugiura T., Uesaka K., Kanemoto H., Mizuno T., Okamura Y. (2013). Elevated preoperative neutrophil-to-lymphocyte ratio as a predictor of survival after gastroenterostomy in patients with advanced pancreatic adenocarcinoma. *Annals of Surgical Oncology*.

[15] Garcea G., Ladwa N., Neal C. P., Metcalfe M. S., Dennison A. R., Berry D. P. (2011). Preoperative neutrophil-to-lymphocyte ratio (NLR) is associated with reduced disease-free survival following curative resection of pancreatic adenocarcinoma. *World Journal of Surgery*.

[16] Templeton A. J., McNamara M. G., Šeruga B. (2014). Prognostic role of neutrophil-to-lymphocyte ratio in solid tumors: a systematic review and meta-analysis. *JNCI: Journal of the National Cancer Institute*.

[17] Guthrie G. J. K., Charles K. A., Roxburgh C. S. D., Horgan P. G., McMillan D. C., Clarke S. J. (2013). The systemic inflammation-based neutrophil–lymphocyte ratio: experience in patients with cancer. *Critical Reviews in Oncology/Hematology*.

[18] Shimada H., Takiguchi N., Kainuma O. (2010). High preoperative neutrophil–lymphocyte ratio predicts poor survival in patients with gastric cancer. *Gastric Cancer*.

[19] Tomita M., Shimizu T., Ayabe T., Yonei A., Onitsuka T. (2011). Preoperative neutrophil to lymphocyte ratio as a prognostic predictor after curative resection for non-small cell lung cancer. *Anticancer Research*.

[20] Ohno Y., Nakashima J., Ohori M., Gondo T., Hatano T., Tachibana M. (2012). Followup of neutrophil-to-lymphocyte ratio and recurrence of clear cell renal cell carcinoma. *The Journal of Urology*.

[21] Chen J., Deng Q., Pan Y. (2015). Prognostic value of neutrophil-to-lymphocyte ratio in breast cancer. *FEBS Open Bio*.

[22] Orditura M., Galizia G., Diana A. (2016). Neutrophil to lymphocyte ratio (NLR) for prediction of distant metastasis-free survival (DMFS) in early breast cancer: a propensity score-matched analysis. *ESMO Open*.

[23] Wang D. S., Luo H. Y., Qiu M. Z. (2012). Comparison of the prognostic values of various inflammation based factors in patients with pancreatic cancer. *Medical Oncology*.

[24] Luo G., Guo M., Liu Z. (2015). Blood neutrophil-lymphocyte ratio predicts survival in patients with advanced pancreatic cancer treated with chemotherapy. *Annals of Surgical Oncology*.

[25] Martin H. L., Ohara K., Kiberu A., Van Hagen T., Davidson A., Khattak M. A. (2014). Prognostic value of systemic inflammation-based markers in advanced pancreatic cancer. *Internal Medicine Journal*.

[26] Verweij J., Therasse P., Eisenhauer E., RECIST Working Group (2009). Cancer clinical trial outcomes: any progress in tumour-size assessment?. *European Journal of Cancer*.

[27] Kaplan E. L., Meier P. (1958). Nonparametric estimation from incomplete observations. *Journal of the American Statistical Association*.

[28] Yang J. J., Hu Z. G., Shi W. X., Deng T., He S. Q., Yuan S. G. (2015). Prognostic significance of neutrophil to lymphocyte ratio in pancreatic cancer: a meta-analysis. *World Journal of Gastroenterology*.

[29] Goldstein D., El-Maraghi R. H., Hammel P. (2015). Nab-paclitaxel plus gemcitabine for metastatic pancreatic cancer: long-term survival from a phase III trial. *JNCI Journal of the National Cancer Institute*.

